# In-Cylinder Pressure Estimation from Rotational Speed Measurements via Extended Kalman Filter

**DOI:** 10.3390/s23094326

**Published:** 2023-04-27

**Authors:** Renato Quartullo, Andrea Garulli, Antonio Giannitrapani, Ryota Minamino, Giovanni Vichi

**Affiliations:** 1 Dipartimento di Ingegneria dell’Informazionee Scienze Matematiche, Università di Siena, 53100 Siena, Italy; quartullo@diism.unisi.it (R.Q.); giannitrapani@diism.unisi.it (A.G.); 2 Yanmar Holdings Co., Ltd., Osaka-shi 8308311, Japan; ryota_minamino@yanmar.com; 3 Yanmar R&D Europe, 50125 Firenze, Italy; giovanni_vichi@yanmar.com

**Keywords:** in-cylinder pressure estimation, Kalman filter, internal combustion engine

## Abstract

Real-time estimation of the in-cylinder pressure of combustion engines is crucial to detect failures and improve the performance of the engine control system. A new estimation scheme is proposed based on the Extended Kalman Filter, which exploits measurements of the engine rotational speed provided by a standard phonic wheel sensor. The main novelty lies in a parameterization of the combustion pressure, which is generated by averaging experimental data collected in different operating points. The proposed approach is validated on real data from a turbocharged compression ignition engine, including both nominal and off-nominal working conditions. The experimental results show that the proposed technique accurately reconstructs the pressure profile, featuring a fit performance index exceeding 90% most of the time. Moreover, it can track changes in the engine operating conditions as well as detect the presence of cylinder-to-cylinder variations.

## 1. Introduction

The estimation of the in-cylinder pressure of internal combustion engines plays a key role within monitoring and control systems to ensure operation efficiency, regulate emissions and detect possible engine malfunctioning [1,2,3,4,5]. Direct measurements provided by dynamic pressure sensors are clearly a possible solution; see, for example, [6,7,8,9]. However, this is usually avoided in production engines for both technological reasons (difficult calibration, long-term reliability issues) and cost limitations. Hence, several indirect methods for reconstructing the pressure profile have been considered in the literature. They differ for the estimation techniques adopted as well as for the measured quantity, including vibration signals [10], acoustic emissions [11], and mechanical stress [12].

Most of the proposed methods have in common the use of measurements of the engine speed and the reconstruction of the in-cylinder pressure from an estimate of the indicated torque. The first seminal works in this respect are based on frequency domain signal processing [13,14]. In [13], a model for the dynamics of an internal combustion engine is proposed, in which the input is given by the cylinder pressure and the outputs correspond to crankshaft angular acceleration and crankshaft torque. In [14], a deconvolution method for the estimation of the mean torque produced by each cylinder during each stroke, from a measurement of crankshaft angular velocity, is proposed. In [15], the authors estimate a linear correlation between the in-cylinder pressure and the engine speed by using frequency response functions.

An alternative line of research is that of model-based estimation methods. In [16,17], the authors combine the speed signal with the measurement of the pressure in one key cylinder to reconstruct the indicated torque and hence the pressure in every cylinder. A drawback of this approach is to require the presence of a pressure sensor in at least one cylinder, thus, involving more expensive and invasive solutions, as well as a higher deterioration risk. Estimation methods based on sliding observers have been proposed in [18,19]. Nonlinear models, such as NARX, recurrent neural networks, and Chebishev polynomial expansions, have been also considered [20,21,22,23]. An approach based on the combined use of crankshaft speed measurements and a zero-dimensional (0D) thermodynamic model has been recently presented in [24].

Another family of approaches for pressure estimation is based on Kalman filtering. Such techniques require a state-space model representation of the overall system, in which the state vector may include both variables of interest and model parameters to be estimated. The works [25,26,27] share a common framework in which the indicated torque is estimated from measurements of the engine speed, and the squared engine speed is chosen as a state variable. In [25], the authors choose directly the net torque as an additional state variable, modeling its dynamics as a discrete low pass filter excited by a white process. The in-cylinder pressure is then reconstructed from the net torque estimate provided by a Kalman filter. A similar approach is taken in [26], in which the periodicity of the system is exploited to set up a time-varying Kalman filter. In [27], the indicated torque is decomposed in two terms, due, respectively, to the compression and combustion pressure, both provided by a 0D cylinder pressure model. The compression term is assumed to be equal for all cylinders, while the shape of the combustion torque of each cylinder is scaled and shifted in the crank angle domain to increase the flexibility in modeling the combustion pressure. This approach is further enhanced in [28], where the shape of the combustion pressure is provided by a sensor mounted on the first cylinder. A mass-spring-damper model is also added to consider torsional and friction effects. In [20], an extended Kalman filter is employed to train a nonlinear model of the crank kinematics, described by a NARX neural network. Other approaches based on Kalman filtering exploit different sensor measurements. In [29], a Kalman filter is used to smooth the pressure measurements provided by an in-cylinder sensor using a thermodynamic pressure model. Measurements of the engine structural vibrations, provided by an accelerometer on the engine block, are exploited in [30]. In-cylinder pressure estimation based on the mechanical stress measured by a strain washer installed on an engine head screw has been proposed in [31]. Finally, it is worth recalling that there is a vast literature on the estimation of the indicated torque from speed or acceleration measurements; see, for example, [32,33,34], which is closely related to the pressure estimation problem.

In this paper, a method for estimating the in-cylinder pressure from measurements of the crankshaft rotational speed based on an Extended Kalman Filter (EKF) is presented. As in [27], the pressure contributions due to the compression and combustion phases are considered separately. The main novelty of the proposed approach is that the 0D combustion pressure model is replaced by an *experimental parameterization*, obtained by averaging pressure profiles from a set of different operating points. This approach permits better capture of the specific behavior of the engine during the combustion phase in the desired range of operating conditions. Once the parameterization has been established, no in-cylinder pressure sensor is required for online operations. The EKF is employed to estimate the amplitude and phase shift of each cylinder pressure profile. Plugging the estimated parameters into the combustion pressure model, one can finally reconstruct the overall pressure signal. The procedure is validated on real data collected from a Yanmar 4-cylinder 2100cc turbocharged CI Diesel engine. The results of the experimental campaign demonstrate that the proposed technique effectively tracks changes in the engine working conditions. Moreover, it allows one to detect anomalies leading, for example, to cylinder-to-cylinder variations.

The rest of the paper is organized as follows. Section 2 introduces the dynamic model of the engine. In Section 3, the pressure estimation approach based on the EKF is presented. The results of the experimental tests are reported in Section 4, while some concluding remarks are given in Section 5.

## 2. In-Cylinder Pressure Model

In this section, the dynamic model of the engine speed and the parameterization of the pressure signal are detailed.

### 2.1. Engine Speed Dynamics

The net torque at the crankshaft T(φ) is decomposed into three terms as

(1) $$T\left(\varphi \right)=T_{ind}\left(\varphi \right)-T_{load}\left(\varphi \right)-T_{dist}\left(\varphi \right),$$

where $T_{ind}$ is the indicated (or gas) torque, $T_{load}$ is the load torque, $T_{dist}$ is the disturbance torque, whose major component is due to friction, and φ is the crank angle. The net torque is responsible for variations of the kinetic energy, according to the dynamic model [27]

(2) $$T\left(\varphi \right)=\theta \left(\varphi \right)\frac{d\dot{\varphi }}{d\varphi }\dot{\varphi }+\frac{1}{2}\frac{d\theta (\varphi )}{d\varphi }{\dot{\varphi }}^{2},$$

where θ(φ) is the moment of inertia of the crank slider, which depends on the crank angle φ. Notice that θ(φ) and its derivative are known from the geometry and the masses of the pistons.

Equation (2) can be exploited to derive the relationship between the angular speed and the net torque. In fact, inverting (2) and considering the square of the engine speed, one gets

(3) $$\frac{d{\dot{\varphi }}^{2}}{d\varphi }=2\dot{\varphi }\frac{d\dot{\varphi }}{d\varphi }=\frac{2}{\theta (\varphi )}\left(-\frac{1}{2}\frac{d\theta (\varphi )}{d\varphi }{\dot{\varphi }}^{2}+T\left(\varphi \right)\right).$$

Let us write the torque as

(4) $$T=\bar{T}+{\left[T\right]}_{∼}$$

where $\bar{T}$ is the mean torque over one cycle and ${\left[T\right]}_{∼}$ denotes the alternating component of the torque. At steady state the mean $\bar{T}$ is zero, i.e.,

(5) $$\bar{T}={\bar{T}}_{ind}-{\bar{T}}_{load}-{\bar{T}}_{dist}=0.$$

The alternating part of the load torque is negligible over an engine cycle, i.e.,

(6) $${\left[T_{load}\right]}_{∼}≃0.$$

From (1), (4), (5) and (6), one obtains

(7) $$T={\left[T\right]}_{∼}={\left[T_{ind}\right]}_{∼}-{\left[T_{dist}\right]}_{∼}.$$

Exploiting (7), the square engine speed dynamics (3) reads

(8) $$\frac{d{\dot{\varphi }}^{2}}{d\varphi }=\frac{2}{\theta (\varphi )}\left(-\frac{1}{2}\frac{d\theta (\varphi )}{d\varphi }{\dot{\varphi }}^{2}+{\left[T_{ind}\left(\varphi \right)\right]}_{∼}-{\left[T_{dist}\left(\varphi \right)\right]}_{∼}\right).$$

The indicated torque is further decomposed by considering separately the effect of the pressure caused by the compression and the combustion strokes, which will be denoted by $p_{comp}\left(\varphi \right)$ and $p_{cmb}\left(\varphi \right)$, respectively. This leads to

(9) $$T_{ind}\left(\varphi \right)=T_{comp}\left(\varphi \right)+T_{cmb}\left(\varphi \right).$$

The combustion torque of each cylinder is related to the combustion pressure as

(10) $$T_{cmb,l}\left(\varphi \right)=p_{cmb}\left(\varphi_{l}\right)Sh\left(\varphi_{l}\right)$$

where l=1,…,4 is the cylinder index (in firing order), $\varphi_{l}=\varphi -\left(l-1\right)\pi$ is the shifted crank angle of cylinder *l*, *S* is the section of the piston and h(φ) is the crank lever. At this point, the total combustion torque component is given by

(11) $$T_{cmb}\left(\varphi \right)=\sum_{l=1}^{4}T_{cmb,l}\left(\varphi \right).$$

Similarly, the compression torque is equal to

(12) $$T_{comp}\left(\varphi \right)=\sum_{l=1}^{4}T_{comp,l}\left(\varphi \right),$$

where

(13) $$T_{comp,l}\left(\varphi \right)=p_{comp}\left(\varphi_{l}\right)Sh\left(\varphi_{l}\right).$$

### 2.2. Pressure Parameterization

Let us now introduce the parameterization of the in-cylinder pressure to be estimated. As mentioned, the pressure components due to the compression and combustion strokes are treated separately. As in [27], the former is provided by a 0D pressure model without imposed combustion, hereafter denoted by ${\hat{p}}_{comp}$. Since this term depends essentially on the intake manifold pressure, it can be safely assumed to be equal for all cylinders during one engine cycle. Hence, by including (13) into (12), the estimation of the compression torque is

(14) $${\hat{T}}_{comp}\left(\varphi \right)=\sum_{l=1}^{4}{\hat{p}}_{comp}\left(\varphi_{l}\right)Sh\left(\varphi_{l}\right).$$

Conversely, this assumption does not apply to the combustion torque of each cylinder *l*, which may depend on different timing and amount of fuel injected. Consequently, an estimate ${\hat{T}}_{cmb,l}$ of the combustion torque of each cylinder is obtained by replacing $p_{cmb}$ in (10) with a shifted and scaled version of a combustion pressure basis function ${\hat{p}}_{cmb}\left(\varphi \right)$. This leads to

(15) $${\hat{T}}_{cmb,l}\left(\varphi ,\alpha_{l}\right)=\alpha_{1,l}{\hat{p}}_{cmb}\left(\varphi_{l}-\alpha_{2,l}\right)Sh\left(\varphi_{l}\right)$$

where $\alpha_{l}={\left[\alpha_{1,l},\;\alpha_{2,l}\right]}^{T}$, and $\alpha_{1,l}$$\alpha_{2,l}$ denote the scaling and shifting parameters of the *l*-th cylinder, respectively.

In this work, the combustion pressure basis function ${\hat{p}}_{cmb}$ is constructed from real-data of the in-cylinder pressure, directly acquired through an experimental campaign by four Kistler 6125C11 piezoelectric sensors triggered by the angular position registered by an AVL 365C optical encoder keyed on the crankshaft. The pressure trace has been measured in different working conditions of the engine, featuring different loads and rotating speeds, and then averaged by means of a moving window of 70 consecutive acquisition cycles to remove the main noise components. The number of analyzed working points is denoted by $N_{wp}$. It is worth stressing that such data acquisition is performed only once for a given engine, and no pressure sensors are required during normal engine operations. Let us denote by $p_{ref,l}^{i}$ the reference indicated pressure obtained from direct measurements of the pressure in cylinder *l*, with the engine operating in working condition *i*. The combustion pressure basis function is obtained by computing the difference between the reference indicated pressure $p_{ref,l}^{i}$ and the corresponding 0D compression pressure ${\hat{p}}_{comp,l}^{i}$. Then, these signals are averaged over all cylinders and working points, thus, obtaining the combustion pressure basis function

(16) $${\hat{p}}_{cmb}\left(\varphi \right)=\frac{1}{4N_{wp}}\sum_{i=1}^{N_{wp}}\sum_{l=1}^{4}\left[p_{ref,l}^{i}\left(\varphi \right)-{\hat{p}}_{comp,l}^{i}\left(\varphi \right)\right].$$

**Figure 1:**
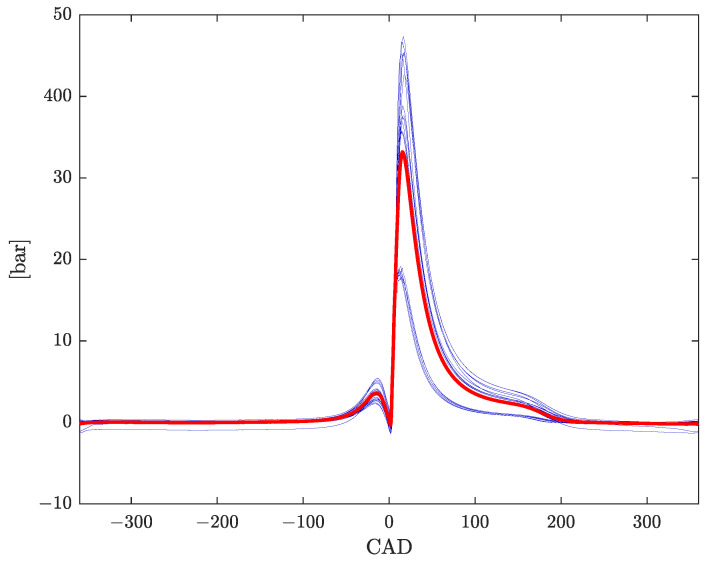
Mean combustion pressure profile ${\hat{p}}_{cmb}\left(\varphi \right)$ (red), computed according to (16) from $N_{wp}=25$ standard condition working points (blue).

Figure 1 displays the average combustion pressure profile, along with the profiles of each working point, resulting from the experimental tests described in Section 4. Note that each combustion pressure profile depicted in Figure 1 (blue curves) is obtained by subtracting the compression profile provided by the 0D model from the measured total in-cylinder pressure. Hence, odd behaviors showing up in some profiles are due to poor estimates of the compression profile by the 0D model.

## 3. Extended Kalman Filter for In-Cylinder Pressure Estimation

In this section, the EKF-based estimation scheme is presented.

The square of the engine speed is set as the first state-space variable of the dynamic model of the EKF, namely $x_{1}={\dot{\varphi }}^{2}$. By using Euler’s method, Equation (8) is discretized with angular step $\Delta \varphi_{0}$, corresponding to the resolution of the toothed flywheel of the angular speed sensor. For ease of notation, let us denote by *k* the discretized angle $k\Delta \varphi_{0}$, with k=0,1,2,…. Then, one gets the equation

(17) $$x_{1}\left(k+1\right)=A_{1}\left(k\right)x_{1}\left(k\right)+A_{2}\left(k\right)T\left(k\right)$$

where

(18) $$\begin{array}{cc} \; & A_{1}\left(k\right)=1+\Delta \varphi_{0}{\left[-\frac{1}{\theta (\varphi )}\frac{d\theta (\varphi )}{d\varphi }\right]}_{\varphi =k\Delta \varphi_{0}}, \end{array}$$

(19) $$\begin{array}{cc} \; & A_{2}\left(k\right)=\Delta \varphi_{0}{\left[\frac{2}{\theta (\varphi )}\right]}_{\varphi =k\Delta \varphi_{0}}. \end{array}$$

By plugging the parametric model of the indicated torque (9)–(15) into (17) and including the parameter vector $\alpha ={\left[\alpha_{1}^{T},\ldots ,\;\alpha_{4}^{T}\right]}^{T}$ in the state variables, one gets the extended state space dynamics

(20) $$x\left(k+1\right)=f(x\left(k\right),{\hat{T}}_{comp}\left(k\right))+w\left(k\right),$$

where $x\left(k\right)={\left[x_{1}\left(k\right),\;\alpha^{T}\left(k\right)\right]}^{T}$ is the augmented state vector, the state vector field is given by

(21) $$f\left(x\left(k\right),{\hat{T}}_{comp}\left(k\right)\right)=\left(\begin{array}{c} A_{1}\left(k\right)x_{1}\left(k\right)+A_{2}\left(k\right)\left({\left[{\hat{T}}_{cmb}\left(k,\alpha \left(k\right)\right)\right]}_{∼}+{\left[{\hat{T}}_{comp}\left(k\right)\right]}_{∼}\right)\\ \alpha (k) \end{array}\right)$$

and ${\left[{\hat{T}}_{comp}\left(k\right)\right]}_{∼}$ is treated as a known external input. The disturbance process $w\left(k\right)\in R^{9}$ is modeled as a zero-mean white process with covariance matrix Q, whose choice is a tuning parameter of the filter. The first element $w_{1}\left(k\right)$ accounts for all unmodeled effects in the speed dynamics, including the alternate component of the disturbance load ${\left[T_{dist}\left(k\right)\right]}_{∼}$. The remaining components $w_{i}\left(k\right)$, i=2,…,9 are used to tune the bias-variance trade-off in the estimate of the parameters α.

The alternating part of ${\hat{T}}_{comp}\left(k\right)$ is computed by simply subtracting the mean value, averaged on an entire engine cycle. This is possible because the knowledge of the compression pressure coming from the 0D model is always available online during the engine cycle. For the combustion torque, at each filtering step *k*, the mean component is computed through the model (11)–(15) with the current estimate of the parameter vector $\hat{\alpha }\left(k\right)$, thus

(22) $${\bar{T}}_{cmb}\left(\hat{\alpha }\left(k\right)\right)=\frac{1}{N_{s}}\sum_{k=0}^{N_{s}-1}\sum_{l=1}^{4}{\hat{T}}_{cmb,l}\left(\varphi ,\hat{\alpha }\left(k\right)\right),$$

where $N_{s}=\frac{720}{\Delta \varphi_{0}}$ is the number of samples in a single engine cycle. At this point, the oscillating combustion torque reads

(23) $${\left[{\hat{T}}_{cmb}\left(k,\hat{\alpha }\left(k\right)\right)\right]}_{∼}={\hat{T}}_{cmb}\left(k,\hat{\alpha }\left(k\right)\right)-{\bar{T}}_{cmb}\left(\hat{\alpha }\left(k\right)\right).$$

It can be observed that the state dynamics (20) is not linear due to the presence of the phase shift $\alpha_{2,l}$ in the combustion pressure profile ${\hat{p}}_{cmb}$ in (15). This is the reason why the EKF is adopted to estimate the state x(k) by using measurements of the squared engine speed. Once the parameter estimates ${\hat{\alpha }}_{l}$, l=1,⋯,4, are available, an estimate of the in-cylinder pressure for cylinder *l* can be obtained as

(24) $${\hat{p}}_{l}\left(\varphi ,{\hat{\alpha }}_{l}\right)={\hat{p}}_{comp}\left(\varphi_{l}\right)+{\hat{\alpha }}_{1,l}{\hat{p}}_{cmb}\left(\varphi_{l}-{\hat{\alpha }}_{2,l}\right).$$

Instantaneous engine speed measurements

(25) $$y\left(k\right)=h\left(x\left(k\right)\right)+v\left(k\right)=\sqrt{x_{1}\left(k\right)}+v\left(k\right)$$

are fed to the EKF. The measurement error, denoted by v(k) in (25), is modeled as a zero-mean white process with variance *R*.

Let $\hat{x}\left(k|k\right)$ denote the estimate of the state x at time *k* based on the observations y(h) of the output, for h=0,…,k and P(k|k) be its covariance matrix. Then, the standard equations of the EKF prediction step are [35]

(26) $$\begin{array}{ccc} \hat{x}\left(k+1|k\right) & = & f(\hat{x}\left(k|k\right),{\hat{T}}_{comp}\left(k\right)) \end{array}$$

(27) $$\begin{array}{ccc} P(k+1|k) & = & F\left(k\right)P\left(k|k\right)F{\left(k\right)}^{T}+Q \end{array}$$

while those of the correction step are

(28) $$\begin{array}{ccc} \hat{x}\left(k+1|k+1\right) & = & \hat{x}\left(k+1|k\right)+K\left(k+1\right)\left[y\left(k+1\right)-h(\hat{x}\left(k+1|k\right))\right] \end{array}$$

(29) $$\begin{array}{ccc} P(k+1|k+1) & = & P\left(k+1|k\right)\left[I-H^{T}\left(k+1\right)K^{T}\left(k+1\right)\right] \end{array}$$

where

(30) $$K\left(k+1\right)=P\left(k+1|k\right)H^{T}\left(k+1\right){\left[H\left(k+1\right)P\left(k+1|k\right)H^{T}\left(k+1\right)+R\right]}^{-1}$$

is the Kalman gain. The Jacobian matrix of the vector field in (20) is given by

(31) $$F\left(k\right)={\left[\begin{array}{cc} A_{1}\left(k\right) & F_{1,\alpha }\left(k\right)\\ 0_{8\times 1} & I_{8\times 8} \end{array}\right]}_{\alpha =\hat{\alpha }\left(k\right)},$$

where

(32) $$F_{1,\alpha }\left(k\right)={\left[\frac{\partial f_{1}}{\partial \alpha_{1,l}}\left(k\right),\;\frac{\partial f_{1}}{\partial \alpha_{2,l}}\left(k\right)\right]}_{l=1,\;\ldots ,\;4}\in R^{1\times 8},$$

with

(33) $$\begin{array}{ccc} \frac{\partial f_{1}}{\partial \alpha_{1,l}}\left(k\right) & = & A_{2}\left(k\right){\left[p_{cmb}\left(\varphi -\left(l-1\right)\pi -\alpha_{2,l}\right)Sh\left(\varphi -\left(l-1\right)\pi \right)\right]}_{\varphi =k\Delta \varphi_{0}}, \end{array}$$

(34) $$\begin{array}{ccc} \frac{\partial f_{1}}{\partial \alpha_{2,l}}\left(k\right) & = & -A_{2}\left(k\right){\left[\alpha_{1,l}p_{cmb}^{\prime }\left(\varphi -\left(l-1\right)\pi -\alpha_{2,l}\right)Sh\left(\varphi -\left(l-1\right)\pi \right)\right]}_{\varphi =k\Delta \varphi_{0}}, \end{array}$$

and $p_{cmb}^{\prime }\left(\varphi \right)=\frac{\partial p_{cmb}\left(\varphi \right)}{\partial \varphi }$. The Jacobian of the measurement equation is given by

(35) $$H\left(k+1\right)={\left[\frac{1}{2\sqrt{x_{1}}},\;0_{1\times 8}\right]}_{x_{1}={\hat{x}}_{1}\left(k+1|k\right)}.$$

**Figure 2:**
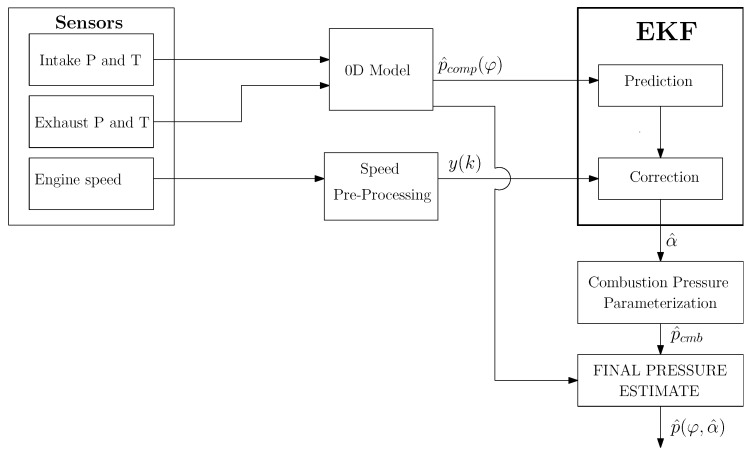
Scheme of the proposed estimation procedure.

The scheme of the overall proposed estimation procedure is outlined in Figure 2. The data about intake and exhaust pressure and temperature, which are usually acquired by the Engine Control Unit (ECU), are employed as boundary conditions in the 0D model integration for obtaining the compression pressure. The raw engine speed measurements, coming directly from the pickup sensor, are suitably pre-processed to compensate for the main torsional effects.

## 4. Experimental Results

**Figure 3:**
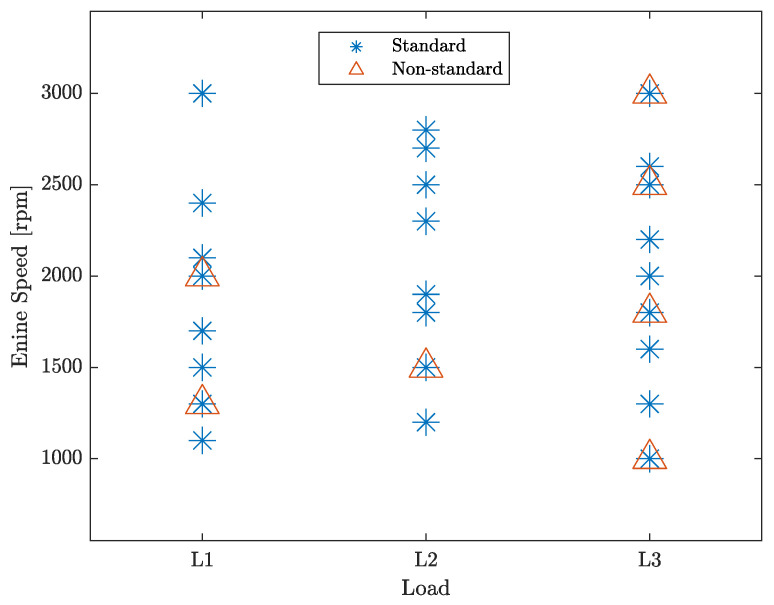
Operating points employed in the experimental campaign expressed in load-rpm coordinates. The working points analyzed only in standard conditions are marked by *, while the ones run also in perturbed engine conditions are flagged with an additional triangular marker.

In this section, the proposed pressure estimation technique is validated on real engine speed data and indicated pressure collected within the experimental test-bed described in [36]. Experimental data have been acquired in a number of working points of a Yanmar 4-cylinder 2100cc turbocharged compression ignition Diesel engine. In particular, such working points feature different loads and speeds. Some tests have been also run under non-standard engine conditions, such as fuel injection anomalies. The different working points are identified by a simple nomenclature. Three different load levels, denoted by L1, L2, and L3, correspond to 10% (low), 50% (medium), and 100% (high) of the maximum engine load, respectively. The engine speed in expressed rpm. The presence of a fuel injection anomaly is indicated by either the letter “n” or “p” plus a number indicating the percentage of under- or over-injection of fuel in the cylinder, respectively. For instance, L1 1300 n5p5p0p0 means that the data are acquired with the engine loaded at minimum, running at a steady state engine speed of 1300 rpm, 5% less fuel is injected in cylinder 1, and 5% more fuel is injected in cylinder 2. All the working points analyzed in both standard and non-standard conditions are displayed in Figure 3. It is worth stressing that they also include working points that were not among those used to construct the basis function ${\hat{p}}_{cmb}$, as described in Section 2.

The results reported hereafter have been obtained by running the proposed method in the Matlab environment on a standard laptop. Moreover, the estimation architecture shown in Figure 2 has been implemented in the ECU of the engine under evaluation, demonstrating that the proposed technique is feasible for real-time operation.

### 4.1. Validation of the Pressure Parameterization

The first set of results concerns the validation of the proposed parameterization of the combustion pressure ${\hat{p}}_{cmb}$. To this aim, a nonlinear least squares error (NLSE) estimate of the optimal parameter pairs $\alpha_{l}^{*}=\left[\alpha_{1,l}^{*},\alpha_{2,l}^{*}\right]$ for each cylinder *l*, has been computed. This amounts to minimize the error between the estimated pressure (24) and the reference pressure $p_{ref,l}$, hence

(36) $$\alpha_{l}^{*}=\arg\min_{\alpha }\left|\right|{\hat{p}}_{l}\left(\varphi ,\alpha \right)-p_{ref,l}{\left|\right|}^{2},$$

where the norm operator indicates the signal $L_{2}$-norm. This method has been applied for both the parameterization ${\hat{p}}_{cmb}$ proposed in this paper and for the combustion pressure profile obtained by the 0D model with imposed combustion, namely ${\hat{p}}_{cmb,0D}$, which is used in [27].

**Figure 4:**
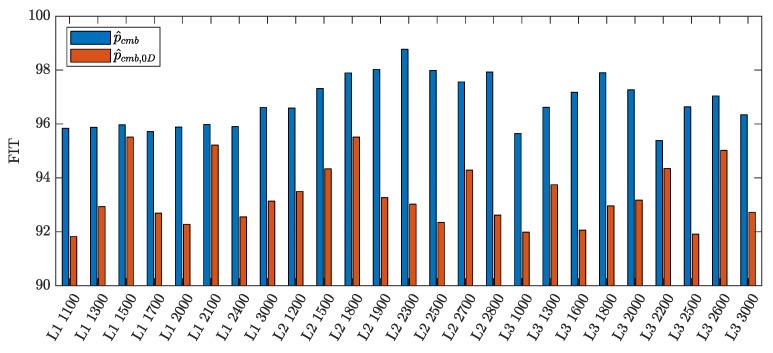
Comparison of the mean FIT, computed as in (37) and (38), of NLSE estimates obtained using the proposed parameterization ${\hat{p}}_{cmb}$ (blue) and the 0D model parameterization ${\hat{p}}_{cmb,0D}$ (red) in problem (36).

Let us define the FIT as a pressure estimation performance index as follows:

(37) $$\mathrm{FIT}=\frac{1}{4}\sum_{l=1}^{4}{\mathrm{FIT}}_{l}$$

with

(38) $${\mathrm{FIT}}_{l}=100\left(1-\frac{\left|\right|p_{ref,l}-{\hat{p}}_{l}\left|\right|}{\left|\right|p_{ref,l}-{\bar{p}}_{ref,l}\left|\right|}\right),$$

where ${\bar{p}}_{ref,l}$ is the sample mean of the reference pressure in cylinder *l*. The average FIT among the cylinders of each selected working point is used as a benchmark for comparison. As shown in Figure 4, the mean FIT of the in-cylinders pressure obtained by the proposed experimental parameterization is always higher than the one relying on the combustion pressure returned by the 0D model. Although these results have been obtained offline, they suggest that the proposed parameterization is promising for combustion pressure fitting and, thus, for indicated pressure estimation.

### 4.2. EKF Pressure Estimation

The engine speed has been measured by a phonic wheel sensor with 60-2 teeth. The discretization has been enhanced using cubic spline interpolation, providing 1440 points per cycle from the original 116. This corresponds to a sampling angular step of $\Delta \varphi_{0}=0.5$ CAD. A moving average filter then smoothes this signal to reduce the measurement noise. The main torsional effects are compensated by an additional pre-stored correction vector and then further corrected in the frequency domain exploiting a 0D engine model with imposed combustion, as detailed in [36]. Such processed measurements are then fed to the EKF presented in Section 3. The filter is run for 300 consecutive engine cycles, which have been observed to be fully sufficient to achieve convergence of the parameter estimates.

The process disturbance covariance matrix and the covariance of noise *v* are chosen after a trial and error tuning approach and set equal to $Q=\mathrm{diag}\{1.6\times 10^{5},\;5\times 10^{-9},\;1\times 10^{-6},\ldots ,\;5\times 10^{-9},\;1\times 10^{-6}\}$ and R=1, respectively. The initial state estimate and its covariance matrix for the filter iteration are set equal to $\hat{x}\left(0|-1\right)={\left[2000^{2},\;1,\;0,\ldots 1,\;0\right]}^{T}$ and $P\left(0|-1\right)=\mathrm{diag}\left\{2.5\times 10^{7},\;2\times 10^{-2},\;1\times 10^{-2},\ldots 2\times 10^{-2},\;1\times 10^{-2}\right\}$.

**Figure 5:**
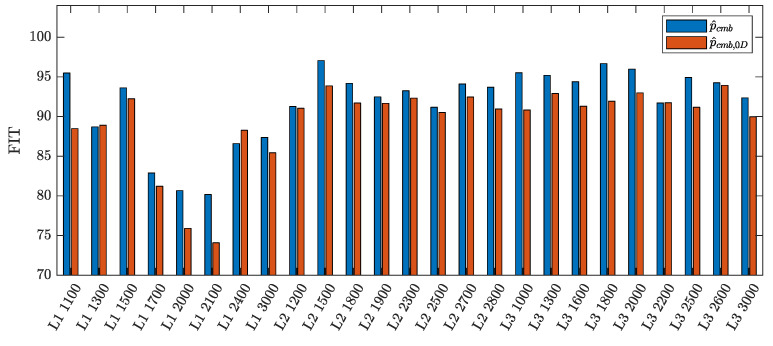
Comparison of the mean FIT of EKF estimates obtained using the proposed parameterization ${\hat{p}}_{cmb}$ (blue) and the 0D model parameterization ${\hat{p}}_{cmb,0D}$ (red), for standard working points.

**Figure 6:**
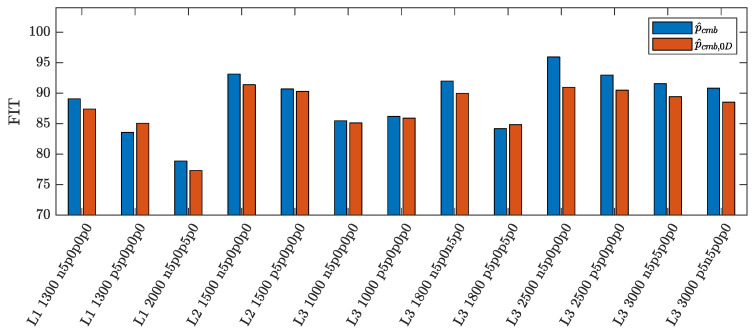
Comparison of the mean FIT of EKF estimates obtained using the proposed parameterization ${\hat{p}}_{cmb}$ (blue) and the 0D model parameterization ${\hat{p}}_{cmb,0D}$ (red), for non-standard working points.

**Figure 7:**
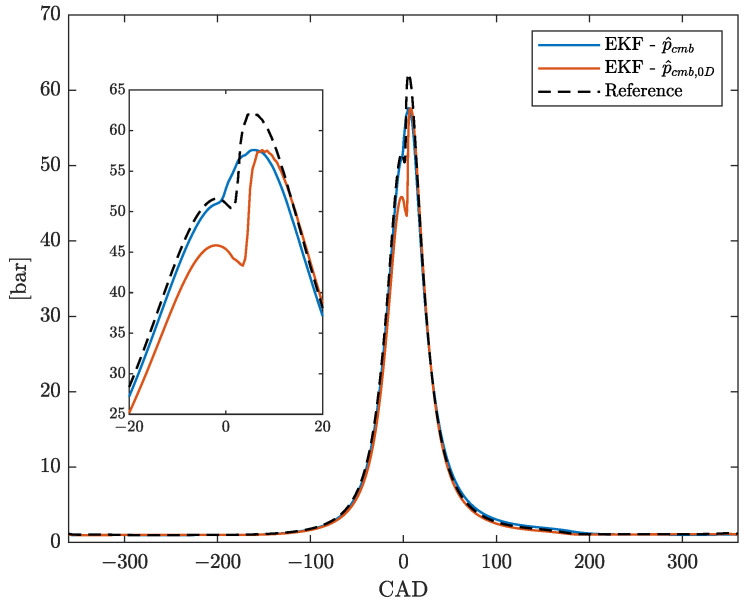
Comparison between the estimates of the total pressure profile, obtained with the parameterizations ${\hat{p}}_{cmb}$ (blue) and ${\hat{p}}_{cmb,0D}$ (red), versus the measured indicated pressure (dashed black), for the first cylinder in the working point L1 1100.

The results in terms of FIT of the steady state EKF estimate of the indicated pressure for all tested points are reported in Figure 5 and Figure 6, together with the ones obtained by using ${\hat{p}}_{cmb,0D}$ as basis function, according to what done in [27]. First, it can be seen that the FIT reached by the proposed EKF scheme is very high, meaning that the estimated pressure profiles fit the reference indicated pressure with very good accuracy. Moreover, the proposed parameterization yields better results than the one based on ${\hat{p}}_{cmb,0D}$, except for very few working points. On average, the new parameterization provides a FIT improvement of 2% (considering both standard and non-standard working points). An example of such improvement is shown in Figure 7, which depicts the pressure estimates of the EKF with both the proposed parameterization ${\hat{p}}_{cmb}$ and ${\hat{p}}_{cmb,0D}$ together with the measured indicated pressure of the first cylinder, i.e., $p_{ref,1}$, of the standard working point L1 1100.

Similar results have also been achieved in terms of IMEP. Table 1 shows some statistics about the IMEP estimation error using the two parameterizations under discussion, with respect to the one computed on the reference pressure $p_{ref}$. It can be seen that, even though the proposed methodology is not specifically designed for this specific performance index, it achieves good accuracy also as an IMEP estimator.

**Figure 8:**
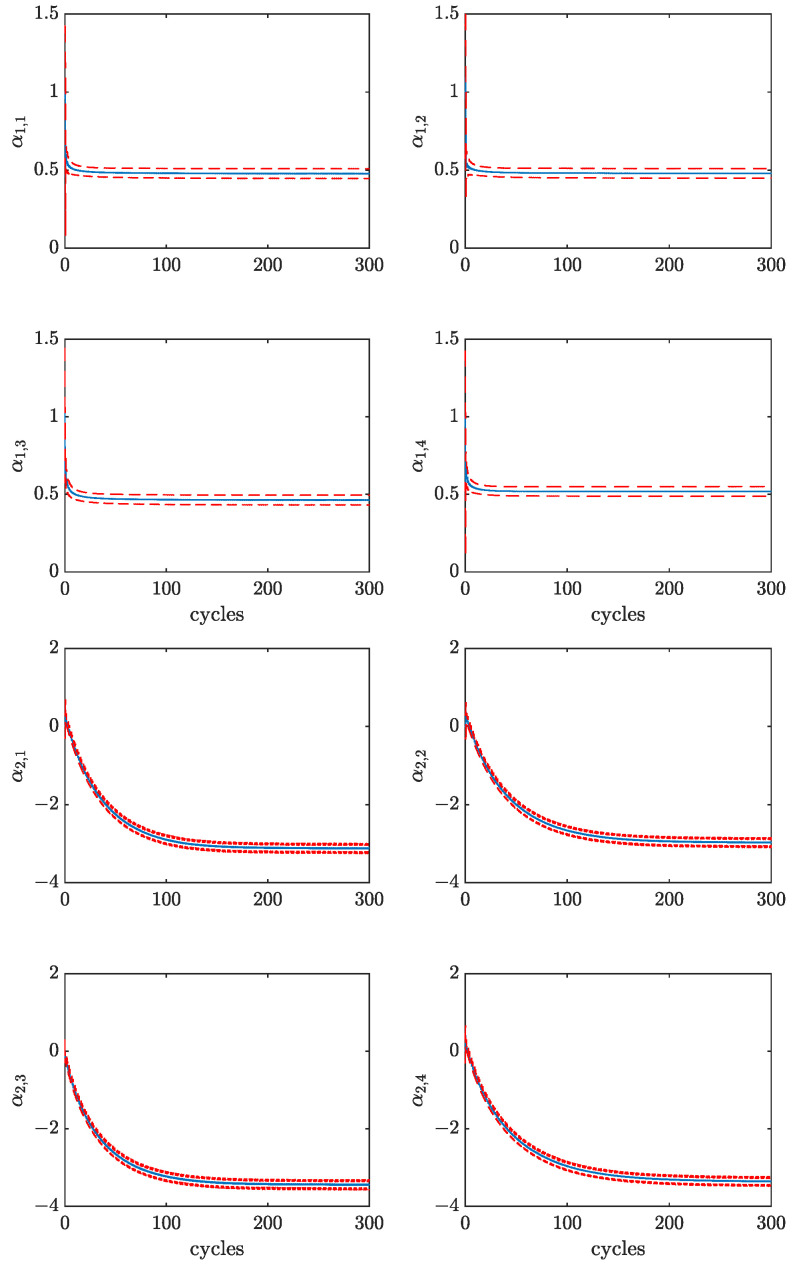
EKF estimates of parameters $\alpha_{1}$ (**top**) and $\alpha_{2}$ (**bottom**) of all cylinders for working point L1 1100 with confidence intervals.

In Figure 8, the estimates of the parameters α are reported together with their 3σ-confidence intervals, for the same working point. It can be observed that all parameters converge to a steady state value in approximately 100 cycles, corresponding to a few seconds. Notice that the scaling parameters $\alpha_{1}$ have a much faster convergence than the shifting ones $\alpha_{2}$. Moreover, the four cylinders show little differences among such parameters since the analyzed working point is run in standard engine conditions.

**Figure 9:**
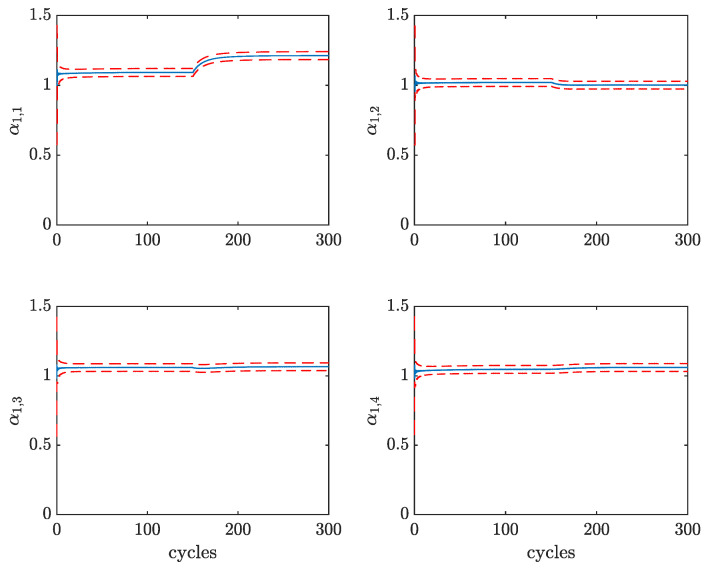
EKF estimates of parameters $\alpha_{1}$ of all cylinders for working point L2 1500 with confidence intervals. After 150 engine cycles, a 5% excess of fuel is injected in the first cylinder so that the new working point is L2 1500 p5p0p0p0.

To validate the behavior of the EKF also during transient engine operations, the working point L2 1500 has been run for 150 cycles, after which a 5% excess of fuel has been injected in the first cylinder, thus, turning the operating conditions into the non-standard working point L2 1500 p5p0p0p0. The evolution of the scaling parameters estimates of this experiment are reported in Figure 9. It can be seen that the parameter $\alpha_{1,1}$ jumps to a higher value, with respect to the other parameters, in less than 50 cycles. This result highlights the adaptability of the proposed approach to engine condition changes and, in particular, the possibility to detect cylinder-to-cylinder variations. Furthermore, by comparing Figure 8 (top) and Figure 9, it can be observed that different loads and engine speeds correspond to different steady-state values of parameter $\alpha_{1}$. For example, the value of parameters $\alpha_{1}$ for the L1 cases is in the order of 0.5, meaning that the combustion pressure profile is a shrunk version of the one used in the proposed parameterization ${\hat{p}}_{cmb}$.

## 5. Conclusions

In this paper, the problem of estimating the in-cylinder pressure of an internal combustion engine from instantaneous engine speed measurements has been addressed. The proposed approach leverages a novel combustion pressure parameterization obtained from experimental data and employs an Extended Kalman Filter to estimate online the pressure parameters. A major advantage of the proposed technique is that direct pressure measurements are used only to construct the data-based parameterization and only a standard phonic wheel speed sensor is employed during normal engine operations.

Extensive experimental validation, covering a wide range of operating points has shown that the proposed approach provides accurate pressure estimates, both in nominal and off-nominal conditions. Overall, it was observed that the novel parameterization is able to better reconstruct the pressure profile. This results in improved fitting performance, when compared to similar techniques relying exclusively on 0D pressure parameterization. A precise reconstruction of the pressure profile turned out to be beneficial also in terms of IMEP estimation. Summing up, the adoption of an Extended Kalman Filter for estimation purposes allow one to automatically adapt the parameter estimates when variations in the operating conditions occur and detects the presence of cylinder-to-cylinder variations at steady-state. The design of filters tailored to the estimation of specific performance indexes (such as IMEP, MFB, etc.) is the subject of ongoing research.

## Figures and Tables

**Table 1:** Statistical results on the IMEP error obtained using the EKF with the proposed parameterization ${\hat{p}}_{cmb}$ and the 0D model parameterization ${\hat{p}}_{cmb,0D}$, for the analyzed standard working points.

	IMEP Error (%)	
	${\hat{p}}_{\mathrm{cmb}}$	${\hat{p}}_{\mathrm{cmb},0D}$
Average	14.8	18.4
Standard Deviation	15.7	21.0

## Data Availability

The data presented in this study are not publicly available due to confidentiality issues.
